# lncRNAs and MYC: An Intricate Relationship

**DOI:** 10.3390/ijms18071497

**Published:** 2017-07-12

**Authors:** Ingram Iaccarino

**Affiliations:** 1 Hematopathology Section, University Hospital Schleswig-Holstein Campus Kiel, Christian-Albrechts University, D-24105 Kiel, Germany; iiaccarino@medgen.uni-kiel.de; Tel.: +49-0431-5001-5721; 2 Institute of Genetics and Biophysics, “A. Buzzati-Traverso”, Consiglio Nazionale delle Ricerche, I-80131 Naples, Italy

**Keywords:** MYC, lncRNAs, transcriptome profiling, cancer, transcription regulation

## Abstract

Long non-coding RNAs (lncRNAs) are emerging as important regulators of gene expression networks, acting either at the transcriptional level, by influencing histone modifications, or at the post-transcriptional level, by controlling mRNA stability and translation. Among the gene expression networks known to influence the process of oncogenic transformation, the one controlled by the proto-oncogene *MYC* is one of the most frequently deregulated in cancer. In B-cell lymphomas, the *MYC* gene is subject to chromosomal rearrangements that result in MYC overexpression. In many other cancers, the region surrounding *MYC* is subject to gene amplification. MYC expression is also controlled at the level of protein and mRNA stability. Neoplastic lesions affecting MYC expression are responsible for a drastic change in the number and the type of genes that are transcriptionally controlled by MYC, depending on differential promoter affinities. Transcriptome profiling of tumor samples has shown that several lncRNAs can be found differentially regulated by MYC in different cancer types and many of them can influence cancer cell viability and proliferation. At the same time, lncRNAs have been shown to be able to control the expression of *MYC* itself, both at transcriptional and post-transcriptional levels. Given that targeting the MYC-dependent transcriptional program has the potential to reach broad anticancer activity, molecular dissection of the complex regulatory mechanisms governing *MYC* expression will be crucial in the future for the identification of novel therapeutic strategies.

## 1. Introduction

The body of complex multicellular organisms is formed by more than 200 different cell types [1]. With the exception of immune cells, in which part of the genome has been rearranged, all these cell types share an identical genome with the same set of protein coding genes. The ability to create the extremely different and functionally diverse cell types that form the body must therefore depend on the ability to differentially regulate the expression of these common genes.

Evolutionary genomics has shown that the number of protein coding genes has not changed dramatically in the transition from simple to complex organisms, despite the striking increase in genome size. There is therefore no correlation between the number of coding genes and organism complexity. On the contrary, organism complexity positively correlates with the proportion of the genome that is dedicated to transcribed non coding genes, that in humans is estimated to be around 70% [2]. Among all non-coding transcripts present in human cells, those classified as long non-coding RNAs (lncRNAs) are emerging as crucial players in the control of gene expression. Recent estimates suggest that up to 70% of all cellular RNAs can be classified as lncRNAs [3]. The analysis of expression profiles across the major human primary cell types and tissues shows the existence of up to 28,000 genes that can be defined as lncRNA [4]. By definition, lncRNAs are RNAs longer than 200 nucleotides and with no predicted coding potential. They can be divided into several subclasses according to their location relative to coding genes (intergenic, intronic, overlapping with exons of coding genes in antisense or sense orientations) [5,6]. lncRNAs have the tendency to be expressed generally at lower level relative to coding genes, but to have higher tissue-specific expression. For some lncRNA loci is the act of transcription that is functionally relevant more than the transcript itself [7]. With the increase in the number of lncRNAs that are functionally investigated, there is also the tentative to apply functional categories to lncRNA classification. For instance, lncRNAs transcribed within enhancers and found to affect enhancer function are called eRNAs [8]. Subcellular localization of lncRNAs is also considered when analyzing lncRNAs as it could indicate a role in epigenetic transcriptional control (strictly nuclear lncRNAs) or in post-transcriptional control (cytoplasmic lncRNAs). Several nuclear lncRNAs were found to be able to regulate chromatin marks either in *cis* or in *trans* by interacting with subunits of chromatin modifying protein complexes [9]. lncRNA can work as guide, using their concomitant ability to fold in specific protein-binding domains and their ability to engage sequence specific base pairing with either DNA or other RNA molecules [10,11]. They can also work as scaffolds in the case of lncRNAs with more protein- or RNA/DNA-binding modules that can bring together different protein complexes or different genomic regions or RNA species [12]. Considering that the genome of a complex organism has genes coding for up to 300 RNA-binding proteins, the combinatorial possibilities of functional ribonucleoprotein complexes engaging lncRNAs is huge. lncRNAs are therefore very likely candidates as molecular players responsible for the increase in gene expression control capabilities associated with the evolution of complex organisms.

The product of the proto-oncogene *MYC* is a subunit of a basic helix-loop-helix and leucine zipper (bHLHZip) heterodimeric transcription factor. Obligate partner for MYC in the assembly of a transcriptional competent complex is another bHLHZip protein called MAX. MAX is able to interact also with other bHLHZip proteins to form complexes that can either activate or repress transcription. The set of genes controlled by the interplay of MYC and MAX with their binding partners is therefore often referred as the MYC transcriptional network [13]. The MYC/MAX complex is able to modulate the transcription of a large number of genes in almost every cell types [14,15,16]. MYC overexpression, either due to chromosomal translocation or to regional gene amplification, is typically associated with an increase in cell growth and proliferation. Accordingly, most of the signaling transduction pathways controlling cellular proliferation converge in the regulation of MYC transcript levels or MYC protein stability. Besides proliferation, MYC has been also implicated in the regulation of genes involved in multiple aspects of physiological changes typical of oncogenic transformation like metabolism, migration/invasion and angiogenesis [17]. Finally high MYC levels are also known to increase cell pluripotency and MYC expression is used to enhance the efficacy of induced pluripotent stem (iPS) cells generation from differentiated somatic cells [18]. Genome-wide analyses have shown that MYC is able to bind to gene promoters and enhancers with different affinities [19]. The number and functional categories of genes controlled by MYC might therefore strongly depend on MYC expression levels: in cells expressing low levels of MYC protein (usually low proliferating cells), mainly high affinity genes will be regulated upon MYC induction. On the contrary, in cells expressing already moderate-high MYC levels, where high affinity binding sites are already saturated, a further MYC increase will result in the regulation of genes having low affinity binding sites in their promoters [19]. Accordingly, constitutive expression of high levels of MYC will result in a general transcriptional amplification [20,21].

In line with the crucial role played by MYC in the definition of cellular transcriptional programs and in line with the role of lncRNAs in the modulation of transcriptional networks in complex organisms, several reports have identified lncRNAs as components of MYC’s transcriptional network. Here, I review the literature describing either lncRNAs found to be regulated by MYC or lncRNA able to affect at any level the activity of the MYC transcriptional network.

## 2. Long Non-Coding RNAs (lncRNAs) Expression Regulated by MYC

As a transcription factor able to influence the expression of a high percentage of all cellular protein coding transcripts, MYC is supposed to influence also the expression of lncRNA transcripts, either directly or indirectly. Already before the advent of next generation sequencing (NGS), the use of tiling arrays coupled with Chromatin Immunoprecipitation (ChIP) showed that MYC was extensively binding outside the regulatory regions of the known protein-coding genes, suggesting it was able to influence the transcription of non-coding genes [22]. Indeed, the group of Penn has found that, by binding to evolutionarily conserved E-boxes, the MYC oncogene can facilitate the transcriptional initiation of *H19*, a lncRNA subjected to genomic imprinting [23]. Later on, studies performed on mouse ES cells identified several intergenic lncRNAs carrying a MYC binding site in their promoter and whose expression was down-regulated following MYC knock-down [24]. In the last three years several groups, including ours, have asked if MYC is able to induce changes in lncRNA expression, by integrating gene expression profiling of MYC-inducible cell lines and MYC ChIP-seq data. The group of Vogt [25] and the group of Kluiver [26] both used the MYC-inducible cell line P493-6, an EBV immortalized B lymphocytic cell line carrying a tetracycline-repressible *MYC* allele. They both found that in P493-6 cells MYC is able to profoundly affect lncRNA transcription, similar to its effect on protein-coding genes. Use of MYC ChIP-seq data obtained from the same cell line, showed a significant enrichment of MYC bound regions in the vicinity of the transcriptions start sites of the lncRNAs regulated by MYC. For some of the identified lncRNAs, the group of Vogt also performed nuclear run-on experiments to show their direct transcriptional regulation by MYC. Overall, these studies show that MYC is indeed able to affect the transcription of many lncRNA genes and that changes in lncRNA expression following MYC activation generally mirrors the changes observed in the transcription of protein coding genes. With the exceptions that MYC induced lncRNAs tends to be more cell-type specific relative to coding genes [25] and that, contrary to MYC down-regulated coding genes (but similar to MYC up-regulated genes) MYC down-regulated lncRNAs are enriched for MYC binding sites in their promoters [26]. 

In P493-6 cells MYC induces a transcriptional program resembling that of Burkitt lymphoma (BL), a B-cell lymphoma characterized by the activating translocation of the *MYC* gene [27]. Accordingly, Winkel et al. found that up to 54% of the lncRNAs identified as MYC regulated in P493-6 cells are regulated also in the comparison between BL and chronic lymphocytic leukemia (CLL), a B-cell malignancy characterized by low MYC levels [26]. Similarly, we carried a differential gene expression analysis comparing BL samples to germinal center-derived normal B-cells [28] and found a strong overlap between BL specific lncRNAs and several of the lncRNAs identified by Hart and collegues in P493-6 cells [25]. Therefore, the MYC-induced transcriptional program strongly influences lncRNA expression in MYC-positive B-cell lymphomas.

Several groups have asked if there are specific lncRNAs regulated by MYC that could play a role in cellular and cancer physiology. Among the validated lncRNAs found by Hart et al., three of them, DANCR, SNHG15 and SNHG16, are genes hosting small nucleolar RNAs (snoRNA) in their introns [25]. Interestingly, we also found several snoRNA hosting genes (including those identified in Hart et al.) up-regulated in BL samples [28]. snoRNAs function is primarily to guide chemical modifications of other RNAs, mainly ribosomal RNAs. Given that ribosomal RNAs rely on RNA editing for a correct structure/function, snoRNAs end up having an important role in ribosomal biogenesis in general. One possibility is that these lncRNAs are upregulated uniquely because of their function as host genes for snoRNAs. This would fit with the view that MYC is considered a master regulator of ribosomal biogenesis [29], due to its ability to control transcription of ribosomal genes [30], snoRNAs [31] and genes coding for ribosomal proteins [32]. On the other hand, Hart et al. showed that at least some of them (DANCR and SNHG16) have a clear cytoplasmic location, suggesting that these genes may have a snoRNA-independent function once exported in the cytosol. A possible snoRNA-independent function was already suggested for DANCR in the process of epidermal differentiation [33]. If DANCR or other snoRNA hosting genes have a snoRNA-independent function also downstream of MYC activation needs to be established.

To identify MYC-regulated lncRNAs that are also regulated in MYC-positive lymphomas and may therefore be functionally relevant for lymphoma development, our group performed an integrated analysis of RNA-seq data from a large cohort of B-cell derived lymphoma samples as well as normal B-cells and compared them with expression data from hT-RPE-MycER cells, an epithelial cell line carrying an activatable MYC protein, as well as to the expression data produced by Vogt group in P493-6 cells [34]. This analysis led to the identification of a lncRNA that we named MINCR (MYC-induced long non coding RNA) [28]. MINCR is up-regulated by MYC in both model cell lines and in all B-cell lymphomas carrying a *MYC*-translocation resulting in MYC overexpression. Furthermore the *MINCR* gene has a MYC binding site at the transcription start site in several cell lines, including BL cell lines and P493-6 cells, and its transcription level goes down after MYC knock-down in BL cell lines [28]. *MINCR* is an intergenic lncRNA lying between two genes coding for the zinc finger proteins GLI4 and ZNF696. Promoter and gene body of *MINCR* are characterized by open chromatin typical of an active promoter in all cell lines analyzed in the context of the Encyclopedia of DNA Elements (ENCODE). Accordingly MINCR is relatively highly expressed in all ENCODE cell lines. An analysis of public ChIA Pet data shows that the promoter region of *MINCR* is engaged in physical contacts with the promoters of *GLI4* and *ZNF696*, suggesting that the three genes may be co-regulated. Although the precise function of MINCR has not been fully elucidated yet, we found clear evidence that MINCR is able to control normal cell cycle progression and it is interfering with the ability of MYC to transactivate a set of genes involved in cell cycle progression. Accordingly the group of Quan found that MINCR knock-down was able to strongly reduce the ability of gallbladder cancer cells to form tumors in nude mice [35].

Besides B-cell lymphoma, the oncogene *MYC* is known to be overexpressed also in colorectal cancer (CRC), where it plays an important role in tumor progression downstream of the WNT signaling pathway [36]. To identify lncRNAs that are deregulated in CRC, the group of Croce employed a lncRNA microarray to profile lncRNAs in normal colon-derived and CRC-derived cells and tissues [37]. Among the CRC-specific lncRNAs, those that are MYC-regulated were selected by looking at lncRNAs concomitantly down-regulated following MYC knock-down in two different CRC cell lines. This strategy led to the identification of three lncRNAs upregulated by MYC in CRC cell lines: AK021907 (MYCLo-1), ELFN1-AS1 (MYCLo-2 or CCAT6) and KTN1-AS1 (MYCLo-3). Interestingly, KTN1-AS1 was also identified in Hart et al. as a MYC induced gene in P493-6 cells [25] and we found it also significantly up-regulated by MYC in hT-RPE-MycER cells [28]. At the functional level, the identified lncRNAs were shown to be able to influence cell cycle progression mainly affecting the expression level of cell cycle inhibitors. The same group also identified two lncRNAs that are transcriptionally repressed in CRC cells expressing high MYC levels: AK098037 (MYCLo-4) and LPP-AS2 (MYCLo-5), although their regulation by MYC is presumably indirect [38]. Interestingly, LPP-AS2 seems to be involved in the positive regulation of the growth arrest gene, GADD45A, a gene that is known to be repressed by MYC. Similar to MINCR, therefore, LPP-AS2 could be listed among those MYC regulated lncRNAs that are able to influence the transcriptional activity of MYC itself.

Another lncRNA identified in CRC cell lines as a target of the WNT/MYC axis is VPS9D1-AS1 (MYU) [39]. VPS9D1-AS1 expression is not limited to CRC cell lines but it is a feature also of other cancer cell lines with high MYC expression. Its expression was shown to be controlled directly by MYC, having a MYC binding site in the promoter region that can be bound by MYC and is MYC responsive in reporter assays. Following VPS9D1-AS1 knock-down, CRC cells have a reduced proliferation rate and a reduced ability to form tumors in vivo. Mechanistically, VPS9D1-AS1 is able to enhance the ability of the hnRNP-K protein to stabilize the mRNA of the cyclin-dependent protein kinase CDK6, by interfering with the binding of miR-16 to the 3′UTR of the *CDK6* gene [39].

Searching for lncRNAs that are both transcriptionally regulated by MYC and able to control MYC expression, the group of Wu identified the transcript ENST00000553181.5 (RP11-320M2.1) that they named lncRNA-MIF, for MYC-inhibitory factor. lncRNA-MIF was identified in P493-6 cells as upregulated upon MYC induction. It has three predicted MYC-binding sites in its promoter that were shown to be bound by MYC and responsive to MYC in reporter assays [40]. Interestingly lncRNA-MIF is transcribed divergently to the *ODC1* gene, a known MYC target gene, raising the possibility that the two genes share the same regulatory regions. lncRNA-MIF was shown to influence cellular glycolitic activity by controlling MYC degradation working as a micro RNA (miRNA) sponge.

One more lncRNA reported to be regulated by MYC is the Brain Cytoplasmic RNA 1 (BCYRN1). Although initially identified as brain specific transcript [41], BCYRN1 was later described to be expressed also from several cancer cells, but not from the normal counterpart [42]. Hu and Lu found BCYRN1 up-regulated in non-small-cell lung cancer (NSCLC) in a MYC dependent manner and to be involved in cell migration and invasion [43]. A list of the MYC-regulated lncRNAs described so far and those described later can be found in Table 1.

## 3. lncRNAs Affecting MYC Expression

Contrary to MAX, whose expression is constitutively high in most tissues, the expression of MYC is tightly regulated by mitogenic stimulation. The amount of active MYC/MAX transcriptional complex is therefore highly dependent on changes in the expression levels of MYC. At least two proximal promoters, P1 and P2, are responsible for *MYC* transcription regulation and are used in normal cells by a multitude of mitogenic stimuli differing form cell type and developmental stage [44]. Far upstream elements have also been implicated in *MYC* transcriptional control [45]. MYC expression levels are not only regulated transcriptionally but also at the post-transcriptional level, by controlling mRNA stability and protein translation and degradation. The fast turnover of MYC is driven by at least two ubiquitin ligases, FBXW7 and SKP2 [46,47,48]. More recently several micro RNAs (miRNAs) have been shown to be able to bind MYC 3′UTR and inhibit its translation or increase the degradation of its mRNA [49].

The *MYC* gene is known to lie in a so-called “gene desert”, a chromosomal region characterized by extremely few genes. This observation holds true if we focus on protein coding genes, but the same cannot be asserted if in the concept of gene we include also non-protein coding genes [50]. Indeed, several lncRNA as well as miRNA genes are found upstream and downstream of the *MYC* gene both in human and in mouse genomes. Functional studies are revealing that many of these non-coding genes have an important role in the regulation of MYC expression.

Studies on lymphomas and leukemias carrying rearrangements in the *MYC* locus have been extremely useful for the understanding of the role of the regions adjacent to *MYC* in the control of MYC transcriptional regulation. In Burkitt Lymphomas, the *MYC* gene is reciprocally rearranged with the heavy or the light chains of the immunoglobulin (Ig) gene loci. The most common translocations with the heavy Ig chain fall either in the first exon or in the first intron of the MYC gene. In some more rare variants the Ig light chain translocates to a 4.5 kb cluster located at least 40–45 kb downstream of *MYC*, the so-called plasmacytoma variant translocation (*PVT-1*) region [51]. While the translocations affecting the first exon/intron are thought to disable the control of MYC expression driven by the P1 and P2 promoters (and presumably also from the other upstream region) and put it under the control of the strong Ig enhancer, the reasons for the translocations involving the *PVT-1* region are only recently starting to be elucidated. The *PVT-1* region encodes the lncRNA PVT1, a multi exonic transcript with numerous isoforms that can be also transcriptionally activated by MYC [52]. Interestingly, *PVT1* is found co-amplified with MYC in a high percentage of cancers carrying an amplification of the chromosomal region 8q24.21. Amplification of the *PVT1* gene contributes to the pathophysiology of several cancers, including ovarian and breast [53], prostate [54], mesothelioma [55] and acute promyelocytic leukemia [56]. Fundamental insights in PVT1 function and its relation with MYC came from the group of Bagchi. By creating mice models carrying one extra copy of either *MYC* alone or the region co amplified with *MYC*, including the *PVT1* gene, Tseng and colleagues demonstrated that in these experimental settings only the combination of both *MYC* and *PVT1* is able to drive neoplasia [57]. The reason may lie in the finding that PVT1 has a positive effect on MYC protein stability by protecting MYC from being phosphorylated on T58, an event that is signaling MYC for proteolytic degradation. A similar control of MYC protein level by PVT1 was observed also in acute promyelocytic leukemia cells upon PVT1 knock-down [56]. Accordingly, the CRC cell line HCT116 fail to form tumors in nude mice if the *PVT1* locus is deleted using genome editing [57]. MYC ability to form tumors is therefore strongly dependent on PVT1 expression. On the other hand, even if no increase in tumor formation was found in mice carrying a single extra copy of *PVT1* only [57], PVT1 may have oncogenic properties also independently of MYC. Guan et al. report that PVT1 may exert anti-apoptotic activity and may contribute to tumor development independently of MYC [53]. Furthermore, in the BL variants carrying a translocation at the *PVT-1* locus, the PVT1 transcript was found fused to the constant region of the Ig light chain [58]. It is reasonable to speculate that at high transcription levels PVT1 may acquire other oncogenic properties.

**Figure 1:**
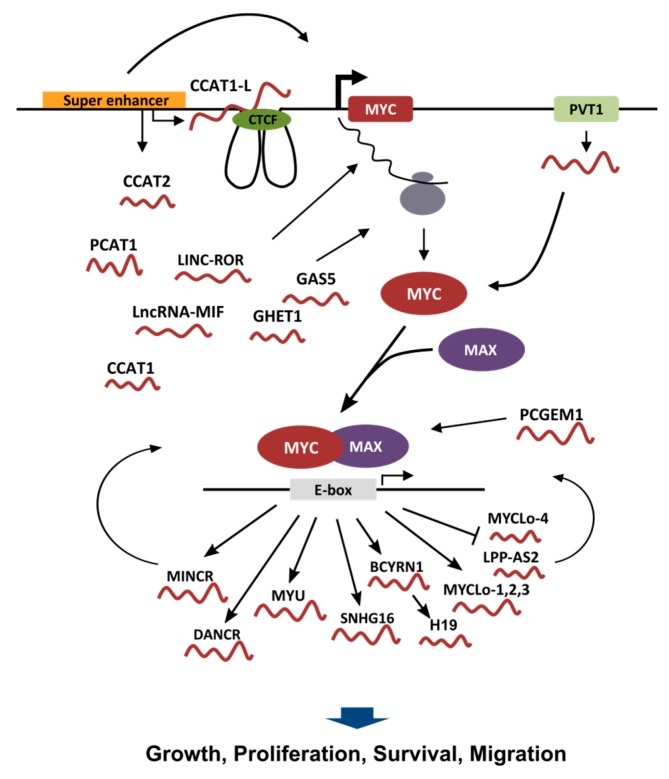
Schematic depiction of lncRNAs involved in the control of MYC transcriptional program. In the upper part, a graphic representation of the *MYC* genomic locus is shown, with the lncRNAs (the “red worms”) involved in the regulation of MYC transcription, MYC mRNA stability, MYC translation and MYC protein stability. Once the MYC protein is formed it heterodimerizes with MAX to form a transcriptional competent factor that binds to the E-box in order to control gene expression. The lower part of the graphic shows the lncRNAs that are transcriptionally regulated by MYC. Some MYC regulated lncRNAs are also able to affect the transcriptional activity of MYC itself. Most of the lncRNAs regulated by MYC have been described to influence growth, proliferation, survival or motility of cancer cells.

Another region playing an important role in the regulation of MYC expression is the region spanning 700 kb upstream of the *MYC* gene. Using H3K27ac ChIP-seq data, the group of Young found a large CRC-specific super-enhancer in this region. The super-enhancer was also cancer specific because absent in the healthy colon tissue [59]. The importance of super-enhancers in the control of MYC expression in cancer is highlighted by the finding that inhibition of BRD4, a transcriptional activator that is particularly enriched in super-enhancers, is very effective in killing cancer cells because it leads to a strong decrease in MYC expression [60,61]. Interestingly, several reports show that super-enhancers are able to produce lncRNAs and that lncRNA transcription may be important for super-enhancer activity [62,63]. In a project aimed at the identification of novel CRC biomarkers, the group of Gure identified Colon Cancer Associated Transcript 1 (CCAT1), a 2628 nt long transcript with consistently strong expression in adenocarcinoma of the colon, and largely undetectable in normal human tissues [64]. The transcript, which was shown to have no coding potential, is located in the CRC-specific super-enhancer region, identified in the study by Hnisz et al., 500 kbp upstream of the *MYC* gene [59]. Interestingly, this transcript (more precisely a longer isoform called CCAT1-L) has been shown to be an important regulator of MYC expression [65]. CCAT1-L is a nuclear retained lncRNA (contrary to the shorter isoform that is cytosolic) whose knock-down reduces MYC transcription level. As shown in Figure 1, CCAT1-L seems to work at the site of transcription by mediating long-range interactions between the *MYC* promoter and the super enhancer through the interaction with the chromatin loop forming factor CTCF [65]. This interaction seems to involve also a region containing the SNP rs6983267, an inherited variant on chromosome 8q24 significantly associated with cancer pathogenesis [66]. The group of Croce also looked for lncRNAs transcribed in the same super-enhancer region and found several Cancer-Associated Region long non-coding RNAs (CARLos). One of those, CARLo-5 is in fact the short isoform of CCAT1 [67]. Knock-down of CCAT1 was found to decrease cell cycle progression by inducing the expression of the cell cycle inhibitor CDKN1A [67]. In this study the authors suggest that the SNP rs6983267 is associated to increased cancer risk because it results in an increase in CCAT1 expression. In synthesis, the CRC-specific super-enhancer is homing at least two lncRNAs sharing one common exon, CCAT1 (CARLo-5) and CCAT1-L. These two lncRNAs may regulate cancer progression in two different ways: either by influencing the tridimensional structure of the locus ending up in increasing MYC expression by bringing the super-enhancer close to the MYC promoter or by controlling the expression of genes related to cell cycle, with a mechanism that is still to be defined. Due to the cytosolic localization of the short isoform of CCAT1 [65], it is reasonable to speculate that a post-transcriptional mechanism may be involved. Interestingly, looking at the ChromHMM Segmentations analysis from the ENCODE Project [68] and at the RNA-seq data from the same cell lines, it is possible to notice that the *CCAT1* locus can have very different activities according to the cell of origin: in the lymphoblastoid cell line GM12878 the locus has marks of a strong enhancer without associated transcript; in the cervical cancer derived HeLa S3 cell line and in the hepatocellular carcinoma derived HepG2 cell line the locus has the marks of an actively transcribed gene with an active promoter and a transcript expressed at high levels; in the CRC derived cell line HCT116 the locus has the marks of a super-enhancer (according to [59]) with the presence of an expressed transcript. 

Two more non-coding transcripts have been identified in the region upstream of *MYC*: CCAT2 [69] and PCAT1 [70]. Both of them have been described to have a pro-oncogenic function and to be associated with MYC expression. CCAT2 was shown to promote cancer growth and metastasis and to increase MYC expression by enhancing TCF7L2 transcriptional activity. PCAT1 was shown to increase cell proliferation of prostate cancer cells by regulating MYC protein at the post-transcriptional level by interfering with miR-34, a miRNA known to target MYC 3′UTR and regulate MYC translation [71]. A graphic depiction of the *MYC* locus with the lncRNAs affecting MYC expression as well as of all other lncRNA described in this review is shown in Figure 1.

Interestingly the mechanism of MYC regulation mediated by lncRNAs working in *cis* is not limited to the *MYC* gene but was found associated also to the *MYC* homolog gene *MYCN*. Amplification of the genomic DNA region containing the *MYCN* oncogene is a recurrent event in almost one fourth of all human neuroblastomas [72]. By asking if there are lncRNAs co-amplified with *MYCN*, the group of Liu identified a non-coding transcript, MYCNUT (lncUSMycN), located 5kb upstream of *MYCN* [73]. MYCNUT knock-down was accompanied by a decrease in MYCN mRNA and protein and its overexpression could increase MYCN mRNA levels. MYCNUT ability to upregulate MYC mRNA expression seems to be dependent on its binding to NONO, a protein known to have a positive effect on mRNA maturation and post-transcriptional processing. Another lncRNA identified upstream of *MYCN* is MYCNOS. Contrary to MYCNUT, MYCNOS is transcribed antisense of MYCN. MYCNOS was shown to function as a modulator of MYCN, able to regulate *MYCN* promoter usage and to recruit various proteins to the upstream *MYCN* promoter [74]. Therefore, similar to *MYC* locus, also the *MYCN* locus is producing lncRNAs that are able to control MYCN expression both at the transcriptional and post-transcriptional level.

## 4. lncRNAs Affecting MYC Stability/Translation

Several other lncRNAs were found to control MYC expression by controlling either the stability of MYC mRNA or the stability/translation of MYC protein. The lncRNA GHET1 was found up-regulated in gastric carcinoma compared to matched non-tumor gastric tissues [75]. Following GHET1 overexpression, the authors observed an increase in MYC levels. Pull down assays demonstrated that GHET1 was able to increase the binding of MYC mRNA to IGF2BP1, a protein able to regulate translation and mRNA stability.

LINC-ROR was originally identified as a lncRNA able to regulate pluripotency in human stem cells [76]. The goup of Mo has asked if LINC-ROR could also play a role in cancer [77]. LINC-ROR was found significantly upregulated in colon cancer tissue relative to normal tissue. Overexpression of LINC-ROR was able to increase tumor growth in xenograft experiments and was accompanied by an increase in MYC expression. At the same time, MYC was strongly reduced in cells with part of the LINC-ROR lncRNA delted. Mechanistically LINC-ROR was shown to affect MYC expression by interacting with two RNA binding proteins: heterogeneous nuclear ribonucleoprotein (hnRNP) I and AU-rich element RNA-binding protein 1 (AUF1). Binding of LINC-ROR to hnRNP I was a prerequisite for hnRNP I to bind to MYC mRNA as well. This interaction results in an increase in mRNA stability. At the same time, LINC-ROR by binding AUF1 prevents this factor from destabilizing MYC mRNA [77].

lncRNAs were found not only regulating MYC mRNA stability, but also MYC protein degradation and mRNA translation. The lncRNA ENST00000553181.5 (lncRNA-MIF) was found to be able to interact with miR-586, a miRNA also able to target the 3′UTR of FBXW7. Accordingly, the authors found that increased expression of lncRNA-MIF increases FBXW7 levels, presumably working as a sponge for miR-586. Given that FBXW7 is a known E3 ligase for MYC, increased FBXW7 is accompanied by an increase in MYC protein degradation [40]. The lncRNA GAS5 on the contrary was suggested to control MYC mRNA translation. Knock-down of GAS5 was accompanied by a strong increase in MYC protein, with no effect on MYC mRNA. The authors found that GAS5 is able to bind to the translation initiation factor eIF4E and at the same time with the MYC mRNA directly. As a result, the overexpression of GAS5 strongly inhibits MYC translation [78].

## 5. lncRNAs Affecting MYC Transcriptional Activity

Long RNA molecules have the peculiar advantage over proteins to potentially act as modular multifunctional platforms. Part of the RNA could fold in a tridimensional structure that could serve as protein-binding domain. At the same time, a second part of the same molecule could use its ability to form complementary RNA/RNA or RNA/DNA hybrid to engage sequence-specific interactions [79]. Based on this concept several lncRNAs have been proposed to work as guides to bring specific proteins on DNA loci or on mRNA molecules. In some cases, the RNA binding proteins are chromatin modifying factors, but there are also examples of transcription factors bound directly by the lncRNA. The prostate cancer gene expression marker 1 (PCGEM1) was initially identified as an androgen-induced prostate-specific lncRNA whose overexpression is highly associated with prostate tumors [80]. More recently, it was shown to regulate tumor metabolism via MYC activation. PCGEM1 was found to physically interact with MYC and enhances its transactivation activity by increasing its ability to bind target promoters [81].

On the same line, we have found that the MYC-induced lncRNA MINCR is able to affect MYC transcriptional activity. Upon MINCR knock-down, we found not only that the genes affected are enriched for MYC target genes, but we also observed a decrease in MYC binding to the promoter of a set of these genes [28]. Although the reason of this observed decrease in DNA binding is still under investigation, we can speculate that MINCR is able to recruit a MYC co-activator to the promoters of some genes, by guiding the co-factor using a sequence specific interaction. Accordingly, we have identified a motif enriched in the promoters of the MINCR controlled genes that could be either a MINCR-bound sequence or the binding site for a cofactor.

It is interesting to speculate that lncRNAs could indeed be the missing “linc” explaining the wide range of binding affinity shown by MYC on the promoters of different genes [19]. Using the sequence complementarity information embedded in a lncRNA a co-factor binding to both MYC and the lncRNA could increase MYC affinity for promoters carrying the complementary motif. Interestingly, the group of Wolf found that the MYC-binding protein WDR5 could strongly influence MYC promoter affinities [19]. As a DNA binding factor, WDR5 can enhance MYC affinity on promoters carrying at the same time MYC binding and WDR5 binding motifs. Interestingly, the group of Chang found that WDR5 has also an RNA binding pocket that is essential for its ability to bind chromatin and to activate gene expression. Importantly, binding to lncRNAs was found crucial for WDR5 to maintain active chromatin and pluripotency in embryonic stem cells [82]. It is reasonable to speculate therefore that WDR5 could influence MYC binding affinity also through lncRNA binding. Furthermore, given the known tissue specificity of lncRNA expression, an involvement of lncRNAs as MYC co-factors could also explain the variability of MYC target genes observed in different tissues and cell types [83].

In conclusion, we are starting to understand that lncRNA may have a crucial role in the correct execution of the gene expression program controlled by the MYC transcriptional network. Due to the pleiotropic effect of MYC expression in oncogenic transformation, MYC is an ideal target for pharmacological intervention, and targeting MYC could have broad-range anti-tumor activity. Unfortunately, MYC has no catalytic activity that can be easily inhibited with small compounds. One alternative approach is to target parallel pathways that converge on MYC regulation or that are required for a proper MYC activity. Therefore, the discovery of novel lncRNAs-mediated pathway that control MYC activity could, in turn, suggest novel therapeutic windows for the fight against all cancer that rely on MYC expression.

## Figures and Tables

**Table 1:** List of lncRNAs (Long Non-coding RNAs) that have been described being part of the MYC network. For each lncRNA, the table shows the official name, alternative names used in the literature, the gene identification number used in the Ensembl database and if the lncRNA is positively or negatively regulated by MYC or it exert a positive or negative effect on MYC expression.

Name	Alternative Name	Ensembl Gene ID	Regulation
lncRNAs regulated by MYC			
H19		ENSG00000130600	positive
DANCR	ANCR	ENSG00000226950.2	positive
SNHG16	NcRAN	ENSG00000163597	positive
MINCR	LINC01604	ENSG00000253716.1	positive
AK021907	MYCLo-1	None	positive
ELFN1-AS1	MYCLo-2	ENSG00000236081	positive
KTN1-AS1	MYCLo-3	ENSG00000186615	positive
AK098037	MYCLo-4	None	negative
LPP-AS2	MYCLo-5,6	ENSG00000270959	negative
VPS9D1-AS1	MYU	ENSG00000261373	positive
ENST00000553181.5	lncRNA-MIF	ENSG00000257135	positive
BCYRN1	BC200a	None	positive
lncRNAs affecting MYC Expression			
PVT1	LINC00079	ENSG00000249859	positive
CCAT1	CARLo-5	ENSG00000247844	unknown
CCAT1-L		ENSG00000247844	positive
CCAT2	LINC00873	ENSG00000280997.1	unknown
PCAT1		ENSG00000253438	positive
MYCNUT	lncUSMycN	ENSG00000223850	positive
MYCNOS		ENSG00000233718	positive
lncRNAs affecting MYC stability/translation			
GHET1		ENSG00000281189.1	positive
LINC-ROR	CTD-2526M8.1	ENSG00000258609	positive
ENST00000553181.5	lncRNA-MIF	ENSG00000257135	negative
GAS5	SNHG2	ENSG00000234741.7	negative
lncRNAs affecting MYC transcriptional activity			
PCGEM1	PCAT9	ENSG00000227418.6	positive
MINCR	LINC01604	ENSG00000253716.1	positive
LPP-AS2	MYCLo-6	ENSG00000270959	negative
